# Educational Needs Analysis for Enhancing Cancer Survivor Nursing Care of Community Nurses

**DOI:** 10.3390/healthcare14142169

**Published:** 2026-07-18

**Authors:** Eun-Jung Bae

**Affiliations:** College of Nursing, Catholic University of Pusan, Busan 46252, Republic of Korea; ejbae@cup.ac.kr

**Keywords:** cancer survivors, education, needs assessment, nurses, public health

## Abstract

**Background/Objectives**: The support of healthcare professionals can affect the improvement of cancer survivors’ self-management ability. Community nurses in charge of primary health care can play a positive role in the management of cancer survivors because they meet with cancer survivors in various areas and have basic knowledge of oncology. This study aimed to assess the educational needs and analyze the priorities of a cancer survivor nursing care education program for community nurses. **Methods**: An online survey was conducted on 164 nurses working in public health centers in South Korea from 5 February to 24 February 2024. A 25-item questionnaire was used to measure community nurses’ perceived knowledge and perceived importance of cancer survivor care. The educational needs analysis was conducted using the Borich needs assessment and the Locus for Focus model. **Results**: The highest priority content areas were as follows: economic support and related insurance systems, second primary cancer screening, physical rehabilitation after cancer treatment, diet and nutrition, complications of cancer treatment, returning to work, physical and mental problems of a cancer patient’s family, and providing training materials and information on cancer. **Conclusions:** The results suggest that community nurses’ educational needs extend beyond direct clinical care to include care coordination and resource-linkage roles. Because these priorities were based on nurses’ perceived educational needs rather than needs grounded in clinical practice, future educational programs should be developed to address these needs while also strengthening community nurses’ clinical reasoning competencies.

## 1. Introduction

Globally, over 35 million new cancer cases are predicted to occur by 2050. As the number of cancer survivors continues to increase owing to earlier detection and advancements in cancer treatment technologies, cancer is considered a major public health issue of the 21st century [1]. In South Korea, the 5-year relative cancer survival rate significantly increased from 42.9% in 1993–1995 to 72.1% in 2017–2021 [2]. Consequently, there is growing interest in the health issues and health-related quality of life of cancer survivors who continue to live after cancer treatment.

Cancer survivors who have undergone cancer treatment often complain of persistent fatigue, pain, sleep disorders, lymphedema, hardships in interpersonal relationships, difficulties in performing roles at the workplace and home, and problems with returning to work [3,4]. Without proper prevention and management of these issues, cancer survivors may encounter increased healthcare costs due to cancer recurrence or metastasis, the development of secondary cancers, and the aggravation of chronic diseases, as well as increased social costs related to social maladjustment and difficulties in returning to daily life [5,6]. Therefore, it is essential to provide cancer survivors with ongoing support and care.

Previously, cancer was recognized as an acute disease; however, recent trends have shifted toward treating it as a chronic disease. To effectively manage chronic diseases, strict self-management is required, and support from healthcare professionals can positively affect the self-management abilities of cancer survivors [7]. However, it was found that healthcare professionals were unable to provide cancer survivors with appropriate support services to address physical, mental, and social problems and received limited education and training with insufficient information and support regarding cancer survivor management [8,9,10,11]. Thus, it is necessary to develop the knowledge, skills, and attitudes of healthcare professionals in managing cancer survivors.

Despite being generally perceived as peripheral to cancer management, primary care has recently gained recognition for its importance in cancer prevention, as well as in the management of survivors and patients with terminal cancer [12]. Public health centers in South Korea are public healthcare institutions established to improve disease prevention, treatment, and public health, and they serve as the foundation of primary care. Community nurses responsible for primary care at health centers, schools, and industrial sites are healthcare professionals who can closely support cancer survivors in various fields. Since they can easily build trust-based relationships with patients and have knowledge of oncology, they can positively affect the self-management of cancer survivors. Additionally, it has been reported that cancer survivors who received nurse-led care had a higher quality of life regarding cognitive and social functions and lower levels of fatigue and appetite loss [13]. Thus, it is necessary to provide systematic nursing care for cancer survivors led by community nurses with sufficient primary care competencies.

In order for community nurses to systematically provide care to cancer survivors, appropriate education must be provided to them. To develop such a curriculum, it is necessary to investigate the differences between perceived knowledge and the perceived importance of nursing care for cancer survivors among community nurses to identify their educational needs. This process serves as the foundation for learner-centered education and is important because it lays the groundwork for defining the scope and priorities of education required to develop an educational program [14,15]. Borich needs assessment and Locus for Focus models are suitable methods for identifying the differences between perceived knowledge and perceived importance to derive the most important educational priorities and define the educational needs of community nurses in cancer survivor care [14,16]. To date, few studies have systematically identified the educational needs of community nurses for cancer survivor nursing using proven methods such as Borich needs analysis and locus for focus models.

Thus, this study sought to identify the perceived knowledge and perceived importance of community nurses in South Korea regarding nursing care for cancer survivors and to define priorities for educational needs, thereby providing basic data for the development of a cancer survivor nursing education program for community nurses.

## 2. Materials and Methods

### 2.1. Study Design

This descriptive survey was conducted to identify the priorities of community nurses’ educational needs for the care of cancer survivors.

### 2.2. Participants

The participants of this study were nurses who had experience providing primary care to cancer survivors in the community and who voluntarily participated in an online survey after reviewing a recruitment announcement that included the goals and descriptions of the study. The sample size was calculated using the G*Power 3.1.9.7 program, applying a confidence level (α) of 0.05, an effect size of 0.25, and a power (1-β) of 0.80 for a paired-sample *t*-test [17], which resulted in a minimum sample size of 128. Considering the dropout rate, the survey was administered to 178 participants. After excluding participants with unreliable responses, 164 were included in the final analysis.

### 2.3. Measurement

The general characteristics of the participants, as well as the knowledge and importance of the content for cancer survivor care education programs, were surveyed using a self-reported questionnaire.

#### 2.3.1. General Characteristics

The general characteristics considered in this study were the participants’ age, education level, total work period as nurses, work period as community nurses, nursing experience for cancer patients in treatment, experience participating in cancer survivor care education, need for cancer survivor care education, and willingness to participate in cancer survivor care education.

#### 2.3.2. The Knowledge and Importance of Each Content for Cancer Survivor Nursing Care Enhancement Programs

To determine the knowledge and importance of each content area of cancer survivor nursing care, the thematic questions developed by Shim et al. [8] for a needs assessment of educational programs for healthcare professionals caring for cancer survivors were used as a survey tool. This tool was developed and reviewed by a multidisciplinary research team consisting of doctors, nurses, and health care policy experts for a national cancer management project in South Korea. The contents of the tool were based on the quality of life model of cancer survivors and The Quality Cancer Care—Declaration of Principles of National Coalition for Cancer Survivorship [18]. The content items of the cancer survivor nursing care enhancement programs were composed of 25 questions covering nine areas, including general introduction to cancer survivors, physical problems, rehabilitation, healthy lifestyle, psychological problems, social problems, spiritual problems, information search and acquisition for cancer survivors, and community networks. The knowledge level for each content area of the cancer survivor nursing care enhancement programs was evaluated using a Likert scale ranging from 1 (completely unfamiliar) to 4 (very familiar), with higher scores indicating higher levels of knowledge among participants. The importance level for each content of the cancer survivor nursing care enhancement program was evaluated using a Likert scale ranging from 1 (not important at all) to 4 (very important), with higher scores indicating higher importance perceived by the participants.

In this study, exploratory factor analysis using principal axis factoring was performed to confirm the construct validity of the tool. Factor extraction was based on eigenvalue 1.0 or higher and oblimin rotation was applied. In the knowledge measurement tool, KMO was 0.95, and the result of Bartlett’s test was statistically significant (χ^2^ = 4187.71, *p* < 0.001). Three factors were extracted, with a cumulative explained variance of 68.5%. All items showed factor loading greater than 0.40, so the entire items were maintained. In the importance measurement tool, KMO was 0.88, and the result of Bartlett’s test was statistically significant (χ^2^ = 2474.25, *p* < 0.001). Five factors were extracted, with a cumulative explained variance of 56.7%. All items were maintained after the factor loading was checked. In this study, Cronbach’s alpha coefficients were 0.97 for knowledge and 0.93 for importance.

#### 2.3.3. Needs for Cancer Survivor Nursing Care Enhancement Education

(1) Borich needs assessment

The Borich needs assessment was utilized to investigate the educational needs regarding a nursing education program on cancer survivor care for community nurses. The needs formula is calculated by multiplying the total difference between knowledge (Present Competency Level, PCL) and importance (Required Competency Level, RCL) by the mean score of importance (by each competency), and then dividing the result by the total number (N). A higher score indicates higher educational needs [14]. In the Borich needs assessment for education, when the importance level perceived by the participants is high but their knowledge is low, the difference between importance and knowledge increases, and thus, educational needs become higher. The formula for calculating educational needs is as follows:

Borich needs = ∑ (RCL − PCL) × MCL/N

RCL = Required Competence Level (Importance), PCL = Present Competence Level (Knowledge), MCL = average score of RCL by each competency, N = total number

(2) Analysis by the Locus for Focus model

The Locus for Focus model was applied to prioritize the educational needs regarding a nursing education program on cancer survivor care for community nurses. This model helps to visually determine which variables should be prioritized using a four-quadrant coordinate plane. The horizontal axis represents importance, while the vertical axis represents discrepancy, measured as the difference between importance and knowledge. The mean importance of all content is positioned at the center of the horizontal axis, and the mean discrepancy is placed at the center of the vertical axis. Each quadrant in this model is divided according to its importance and discrepancy. The first quadrant (high discrepancy/high importance) represents the area with the highest priority, where both discrepancy and importance are higher than the mean. The second quadrant (high discrepancy/low importance) represents the area where the importance is lower than the mean value, but the discrepancy is higher than the mean value, indicating that it is necessary to identify the reasons for the low importance to increase the knowledge level. The third quadrant (low discrepancy/low importance) corresponds to areas where both discrepancy and importance are below the mean values, while the fourth quadrant (low discrepancy/high importance) indicates areas where discrepancy is below the mean value but importance is above the mean value, thereby representing areas with a high level of knowledge [16].

### 2.4. Data Collection

Data was collected through an online survey targeting community nurses from 5 to 24 February 2024. The researcher collaborated with the heads of public health centers in Busan, a recruitment announcement containing the online survey URL was circulated to recruit participants. The participating nurses were instructed to access the online survey URL directly, complete the questionnaire, and submit their responses. On the first screen of the online survey, the purpose of the study and the necessity of providing personal information were explained to the participants. The survey took approximately 15–20 min to complete, and participants were offered a token of appreciation upon completion.

### 2.5. Data Analysis

The data collected in this study were analyzed using the SPSS/WIN 29.0 program (IBM Corp., Armonk, NY, USA). First, the general characteristics of the participants were presented as frequencies, percentages, means, and standard deviations. Second, independent *t*-tests, ANOVA, and Pearson’s correlation coefficients were used to assess discrepancies and correlations between the knowledge and importance of cancer survivor care education content according to general characteristics. Third, paired *t*-tests were used to examine discrepancies between knowledge and importance. Fourth, the Borich needs assessment was used to determine educational needs, overcome the limitation of *t*-tests, which only compare simple differences between knowledge and importance, and identify the priorities of educational needs. Fifth, we visualized the priorities by plotting the results on a coordinate plane using the Locus for Focus model. Finally, we identified the top- and secondary-priority groups by checking the overlap between the top-ranked contents in the Borich needs assessment and the content items in the first quadrant (high discrepancy/high importance) and the second quadrant (high discrepancy/low importance) of the Locus for Focus model.

### 2.6. Ethical Considerations

This study was approved by the Catholic University of Pusan Institutional Review Board (CUPIRB-2022-055). We explained the purpose and methods of the study, the plan for utilizing the collected data, and the risks and compensation associated with participating in the study to all study participants. Additionally, we informed the participants that they could withdraw from the study at any time without any negative consequences. To ensure that only participants who consented to the study were included, an online survey was initiated only for those who agreed to participate in the study. All data and personal information collected were kept anonymous and confidential. Personal identification information collected for the purpose of providing research benefits, such as phone numbers, was permanently deleted immediately upon completion of the survey.

## 3. Results

### 3.1. General Characteristics

The general characteristics of the study participants are presented in Table 1. Most participants were women (92.7%), and the mean age was 43.41 ± 11.37 years, with those in their 30 s (31.1%) accounting for the largest proportion of participants. 93.3% of the participants had graduated from a 3-year or 4-year college or university. The average total work period as nurses was 12.62 ± 8.83 years, with those having 10–20 years of experience accounting for the largest proportion at 32.3%. In addition, the mean length of experience working in the community was 7.68 ± 7.30 years, with nurses with 10 years or more of experience accounting for the largest proportion at 32.9%. However, 52.4% of the participants had no nursing experience for cancer patients in treatment, and 83.5% had no experience participating in education related to nursing care for cancer survivors. However, 99.4% of the participants believed that cancer survivor care education was necessary, and 93.3% expressed their willingness to participate in such education.

In the analysis of the knowledge and importance of cancer survivor care education content according to the general characteristics of community nurses, knowledge level was found to be different depending on age (F = 5.12, *p* = 0.002; r = 0.28, *p* < 0.001), total work period as nurses (F = 2.70, *p* = 0.048; r = 0.21, *p* = 0.007), length of service as community nurses (F = 4.66, *p* = 0.004; r = 0.24, *p* = 0.002), nursing experience for cancer patients in treatment (t = 3.09, *p* = 0.002), experience participating in cancer survivor care education (t = −5.57, *p* < 0.001), and need for cancer survivor care education (t = 2.00, *p* = 0.047). There were no significant differences in the importance of the content of cancer survivor care education according to general characteristics.

### 3.2. Knowledge and Importance by Content of Cancer Survivor Care Education

In terms of knowledge about the content of cancer survivor care education for community nurses, ‘Smoking’ was the highest in the healthy lifestyle category, with a mean score of 3.09 ± 0.69 out of 4 points, followed by ‘Drinking’ in the healthy lifestyle category with 3.05 ± 0.69 points, and ‘Pain’ among physical problems with 3.01 ± 0.64 points. Next were ‘Fatigue’ under physical problems and ‘Distress (depression, anxiety, and stress)’ under psychological problems, both at similar levels of 2.98 ± 0.62 and 2.97 ± 0.68, respectively. On the other hand, ‘Patient organization and related society’ under community network had the lowest score for knowledge (Mean ± SD = 2.15 ± 0.78). Regarding the importance of each content area of cancer survivor care education, ‘Second primary cancer screening’ in healthy lifestyle scored the highest at 3.73 ± 0.46 points, followed by ‘Distress (depression, anxiety, and stress)’ in psychological problems at 3.68 ± 0.50 points, and ‘Diet and nutrition’ and ‘Exercise’ in healthy lifestyle at 3.65 ± 0. 50 and 3.64 ± 0.49, respectively. The lowest content item for importance was found in the ‘Sexual health’’ of physical problems (Mean ± SD = 2.90 ± 0.75) (Table 2).

### 3.3. Analysis of Educational Needs of Caring for Cancer Survivors

To examine the discrepancies in knowledge and importance of cancer survivor care education among community nurses, we conducted a mean difference analysis, which revealed statistically significant discrepancies (*p* < 0.001) in all 25 items. The Borich needs assessment for each content item showed that the need for ‘Economic support and related insurance systems’ scored the highest at 4.02, followed by ‘Patient organization and related society (3.97)’ under community network, ‘Second primary cancer screening (3.79)’ under healthy lifestyle, ‘Physical rehabilitation after cancer treatment (3.76)’ under rehabilitation, and ‘Management of cancer survivors in the community’ under community network (Table 3).

### 3.4. Priority Analysis for Cancer Survivor Care Education

**Figure 1:**
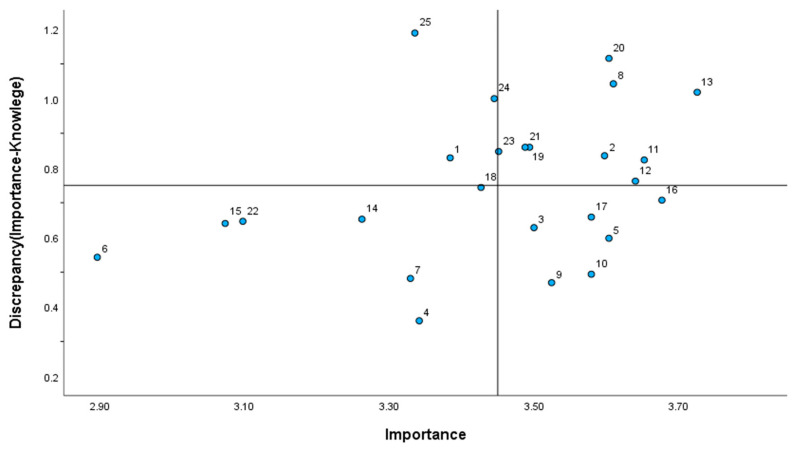
Results for the priority analysis of the educational needs using the Locus for Focus model. (1) Current Status and Key Issues of Cancer Survivors. (2) Cancer treatment complications (3) Management of comorbidities. (4) Fatigue. (5) Pain. (6) Sexual health. (7) Changes in appearance. (8) Physical rehabilitation after cancer treatment. (9) Drinking. (10) Smoking. (11) Diet and nutrition. (12) Exercise. (13) Second Primary Cancer Screening. (14) Vaccinations. (15) Complementary therapy. (16) Distress (depression, anxiety, stress). (17) Sleep disorders. (18) Cognitive decline. (19) Returning to work after treatment. (20) Economic support and related insurance systems. (21) Physical/mental problems in the family of a cancer patient. (22) Religious activities. (23) Provide training materials and information on cancer. (24) Management of cancer survivors in the community. (25) Patient organizations and related societies.

The Locus for Focus model was used to visualize the priorities of the needs for cancer survivor care education among community nurses, as shown in Figure 1. The mean discrepancy between knowledge and importance of cancer survivor care education for community nurses was 0.75, while the mean importance was 3.45. When analyzing the distribution of each content item based on these values, the first quadrant (HH), which had a higher mean value than the mean of the discrepancy between importance and knowledge and also had a higher mean value for educational importance, contained eight content items: ‘Economic support and related insurance systems’, ‘Second primary cancer screening’, ‘Physical rehabilitation after cancer treatment’, ‘Diet and nutrition’, ‘Complications of cancer treatment’, ‘Returning to work’, ‘Physical/mental problems of a cancer patient’s family’, and ‘Provide training materials and information on cancer’. The second quadrant (HL) where the discrepancy was higher than the mean value but the importance level was lower than the mean value, included ‘Patient organization and related society’, ‘Management of cancer survivors in the community’, and ‘Current status and key issues of cancer survivors’. Eight content items were included in both the first quadrant (HH) of the Locus for Focus model and the top 10 contents from the Borich needs assessment as follows (Table 3): ‘Economic support and related insurance systems’, ‘Second primary cancer screening’, ‘Physical rehabilitation after cancer treatment’, ‘Diet and nutrition’, ‘Complications of cancer treatment’, ‘Returning to work’, ‘Physical/mental problems of a cancer patient’s family’, and ‘Provide training materials and information on cancer’.

## 4. Discussion

This study aimed to identify community nurses’ perceived knowledge and perceived importance of cancer survivor care. In addition, we sought to identify priorities in educational needs by applying the Borich needs assessment and the Locus for Focus model to analyze the differences between current perceived knowledge and perceived importance.

Most community nurses who participated in this study had not received any education related to nursing care for cancer survivors (83.5%). In addition, there were differences in their knowledge of nursing care for cancer survivors depending on whether they had received education on the subject. With improvements in cancer survival rates, many cancer survivors now live in communities. Nurses in primary care settings are also more likely to encounter cancer survivors during their work. To provide appropriate nursing care to these cancer survivors, community nurses must improve their knowledge of various nursing needs. In this sense, education on cancer survivors is necessary.

To identify priorities for educational needs, this study identified content items with high priorities in both the Borich needs assessment and Locus for Focus model analysis. The highest-priority content item was ‘Economic support and related insurance systems.’ The high priority means that participants think the importance of the item is high, but they perceive that their knowledge is low. Cancer patients in Korea are subject to the calculation special system for five years after cancer diagnosis under health insurance [19]. Cancer survivors in the long-term survival stage, whose calculation special system has ended, demanded difficulties in maintaining a living due to the burden of long-term treatment costs, support for returning to work, and a rehabilitation program, and expressed them as problems of concern [6]. Because addressing these needs often requires navigating complex financial and welfare systems, community nurses perform tasks such as answering related questions or linking with relevant resources, and in this process, they are highly likely to perceive a lack of knowledge. Therefore, these educational needs suggest that community nurses’ roles are expanding beyond direct clinical care to encompass care coordination and resource-linkage functions, positioning them as facilitators who connect cancer survivors with the broader health and social support network [20]. To provide cancer survivors with appropriate services according to their needs, community nurses must be able to access diverse and substantial community resources. Because community support systems may be registered with local governments, with programs varying by municipality [21], education on procedures and methods for identifying and sharing information about necessary resources should be provided to community nurses. Such education would enable community nurses to provide cancer survivors with broader, more integrated services.

The second priority content item identified was ‘Second primary cancer screening.’ Secondary cancer refers to the development of a new cancer in individuals with a history of cancer, independent of the primary cancer. Because secondary cancer can increase mortality rates among cancer survivors, secondary cancer screening is critical [22]. A study conducted in South Korea found that cancer survivors tended to believe that regular follow-up checks for their primary cancer at hospitals were sufficient to detect anything abnormal in their bodies, leading them to disregard the need for additional periodic cancer screening [23]. Thus, healthcare professionals must be more attentive to this issue and provide appropriate education. In this study, community nurses acknowledged the importance of secondary cancer screening while answering that their related knowledge was low (discrepancy mean = 1.02). Thus, it is necessary to educate community nurses on the importance and methods of secondary cancer screening, enabling them to provide accurate information to cancer survivors.

The priority list of educational needs also included ‘Physical rehabilitation after cancer treatment,’ ‘Diet and nutrition,’ ‘Complications of cancer treatment’, and ‘Provide training materials and information on cancer’, reflecting common treatment-related needs expressed by cancer patients [24]. Most of the community nurses participating in this study had no experience in nursing acute cancer patients (52.4%) and were found to have discrepancies in their knowledge related to nursing cancer survivors depending on whether they had experience for cancer patients in treatment. While nurses recognize that various post-treatment problems may emerge in patients with cancer and that these may cause hardship, they appear to have little confidence in providing appropriate nursing care to patients with cancer. Thus, community nurses need systematic education that include not only basic content, such as treatment methods, complications, and precautions during cancer treatment, but also ongoing methods for searching for and learning about cancer-related materials and information.

For cancer survivors, ‘returning to work’ is an important issue not only for patients themselves but also for social and economic reasons [25], and cancer survivors also have a great demand for information related to various workplace issues [26]. To respond to these demands, nurses should be aware of the support systems that can help cancer survivors return to work after treatment. Cancer causes anxiety and depression not only in survivors but also in family members who care for or live with them, and brings about changes in family relationships and roles, thereby acting as a major source of stress for the family [27]. In a broad sense, cancer survivors include not only those who are diagnosed with cancer but also their family members and friends [28]. Therefore, community nurses who encounter cancer survivors in their daily work are required to expand their knowledge of the item “Physical/mental problems of a cancer patient’s family” and develop the ability to connect them with resources.

Cancer survivors complain of fatigue, pain, sexual health, and cognitive decline [4], but this study found that community nurses perceived not only low knowledge associated with these issues but also low importance. Previous studies have also shown that healthcare provider underestimate the fatigue, pain, and sexual health of cancer patients [29,30]. These problems are subjective areas, and healthcare provider cannot know unless the patient speaks out. In addition, there is no clear intervention manual [31] and it is easy to stay at the level of just listening, which can be pushed back from nurses’ work priorities. From another perspective, the fact that nurses perceive the importance of these issues as low may indicate that they have not been sufficiently addressed within community nursing education curricula and cancer survivorship care guidelines. This can be interpreted as reflecting that existing cancer survivorship education and care systems have primarily focused on physically and clinically visible problems, while relatively subjective, quality-of-life-related domains have been marginalized in both policy and education. In South Korea, the Integrated Support Center for Cancer Survivor is operated for the management of cancer survivors, and a training program for cancer survivorship management experts is also provided [32]. However, existing education programs primarily focus on topics such as cancer diagnosis and treatment methods, health management for cancer patients, management of side effects after cancer treatment, and understanding lymphedema. Moreover, this education is primarily targeted at practitioners working at the Integrated Cancer Survivor Support Center or medical institutions. As such, community nurses are largely excluded from the current cancer survivorship education system, and educational content addressing survivors’ subjective concerns remains insufficiently developed.

A further consideration concerns the relationship between the educational priorities identified in this study and the complexity of problems actually encountered in oncology nursing practice. The priorities identified in this study were derived from nurses’ self-reported perceptions of knowledge and importance, and therefore reflect perceived needs rather than needs grounded directly in clinical practice. This distinction matters because subjective knowledge gaps capture what nurses believe they lack, filtered through the visibility of a problem in routine practice; content areas that are administratively salient may thus be prioritized more readily than problems that are clinically complex but less visible. This gap is illustrated by a recent study analyzing electronic health records of lung cancer patients [33], which identified an average of five nursing diagnoses per patient with comparatively low diagnostic accuracy even among oncology specialist nurses. This suggests that actual care demands are rarely reducible to discrete content domains but instead involve multiple, interacting problems that are difficult to capture through self-report and even more so for community nurses with less concentrated oncology exposure. In other words, subjective knowledge gaps reflect what nurses can readily name as deficient, whereas actual care demands are shaped by patient-level complexity that resists easy self-identification. This implies that the priority list generated here should be paired with training that strengthens clinical reasoning rather than treating each content domain as an independent unit of learning.

In line with this need for more individualized and reasoning-based education, it was additionally analyzed whether there was a difference in educational needs depending on whether the subjects who participated in this study had previously received care training for cancer survivors (Supplementary Materials). Participants with previous educational experience generally perceived that the degree of knowledge in each area was high, but they were different from the priorities of educational needs of subjects who had not previously received education. This may be attributed not only to whether participants had prior educational experience but also to the specific content of the education they received. Therefore, it is necessary to fully consider the general characteristics of the participants and the degree and content of experience of previous education when selecting a topic for education in the future.

Beyond individual clinical reasoning, institutional support is also essential, as cancer survivors in the long-term survival stage have physical, psychological, and social needs, such as fatigue, anxiety, fear of recurrence, social isolation, and uncertainty about their future [3,4,5,6]. The care of nurses has the potential to improve various unmet needs of cancer survivors and ultimately contribute significantly to improving their lives [34]. Oncology Nurse Society’s Oncology Nurse Navigation and the Macmillan Cancer Support model in the UK have integrated around nurses to help cancer survivors’ lives and provide various information to solve various and complex problems [35,36]. Therefore, institutional support is needed as well as efforts to improve the knowledge of individual nurses in the community for the care of long-term life-sustaining cancer survivors.

The tool used in this study to assess community-based nurses’ perceived knowledge and importance was developed based on theoretical review by a group of experts; however, validity verification results for the tool could not be identified. Therefore, exploratory factor analysis was conducted in this study, and the nine domains of the original tool were consolidated into three factors for knowledge and five factors for importance. In the knowledge measurement tool, a single factor encompassed six or more domains, suggesting that participants may have perceived these domains collectively as comprehensive knowledge of overall cancer survivorship care management. This can be interpreted as reflecting that a differentiated knowledge structure was not formed for domains that are not frequently encountered in nursing practice. In the importance measurement tool, most factors were composed of conceptually interrelated items, whereas fatigue, sexual health, changes in appearance, vaccination, and religious activity were combined into a single factor. This result may reflect that participants perceived these items as relatively unfamiliar or assigned them a similarly perceived level of priority. Participants tend to perceive their level of knowledge across domains in a comprehensive manner, whereas they assign importance with more nuanced distinctions across domains. Therefore, future development of the knowledge measurement tool should take into account nurses’ actual frequency of exposure to each domain in practice, and repeated verification with a larger sample, along with confirmatory factor analysis, is needed to establish the stability of the factor structure for both tools.

This study has a few limitations. First, this study measured the perceived degree of knowledge using a structured questionnaire. This was insufficient to identify community nurses’ detailed educational needs regarding nursing care for cancer survivors and to identify the degree of practical knowledge. In the future, interviews or other studies should be conducted to identify specific educational needs, and it is necessary to evaluate the objective level of knowledge and develop customized educational programs accordingly. Second, most participants had not received prior education related to cancer survivor care and varied considerably in their experience of caring for patients undergoing active cancer treatment. This suggests that participants’ perceived knowledge, perceived importance, and the resulting educational priorities may reflect limited prior exposure and education rather than needs grounded in substantial clinical experience. Therefore, caution is needed in interpreting and generalizing the priorities identified in this study, and future research should consider stratifying by prior education and clinical experience to derive more robust findings. Third, this study used convenience sampling of nurses from a single region, making it limited in generalizing the results to all community nurses in South Korea. A more representative sampling method is required to validate the findings of this study. Fourth, the knowledge and importance measurement tools for each content of cancer survivor care used in this study were developed through expert review, and validity and reliability verification for the tools were not conducted. Additional and repeated studies are required to verify the reliability and validity of the tool. Despite these limitations, this study is significant in that it is the study in Korea to analyze the educational needs of community nurses for the care of cancer survivors. In addition, it is expected that the priority education content on cancer survivor care of community nurses identified in this study will be used as foundational data for future curriculum development.

## 5. Conclusions

In this study, we identified the educational needs of community nurses regarding nursing care for cancer survivors and analyzed the priorities of educational content. To determine the priorities of educational content, we utilized the Borich needs assessment and the Locus for Focus model. The results revealed eight educational priorities for nursing care of cancer survivors: ‘Economic support and related insurance systems’, ‘Second primary cancer screening’, ‘Physical rehabilitation after cancer treatment’, ‘Diet and nutrition’, ‘Complications of cancer treatment’, ‘Returning to work’, ‘Physical/mental problems of a cancer patient’s family’, and ‘Provide training materials and information on cancer’. Because these priorities reflect nurses’ perceived educational needs rather than the full complexity of clinical practice, future educational programs should address the identified educational priorities while also strengthening community nurses’ clinical reasoning competencies needed to manage complex survivorship care. Policy support is needed to facilitate the systematic development and continuous implementation of educational programs that enhance both cancer survivorship knowledge and clinical reasoning competencies among community nurses.

## Figures and Tables

**Table 1:** Difference between perceived knowledge and perceived importance of cancer survivor nursing care according to general characteristics (N = 164).

Characteristics	Categories	n (%) or	Knowledge			Importance		
		M ± SD	M ± SD	t/F/r	*p* Scheffé	M ± SD	t/F/r	*p* Scheffé
Gender	Female	152 (92.7)	2.70 ± 0.56	0.15	0.882	3.46 ± 0.36	1.19	0.235
	Male	12 (7.3)	2.68 ± 0.77			3.33 ± 0.35		
Age (years)	20~29 ^a^	16 (9.8)	2.67 ± 0.70	5.12	0.002	3.48 ± 0.33	0.42	0.741
	30~39 ^b^	51 (31.1)	2.53 ± 0.47		b < d	3.41 ± 0.33		
	40~49 ^c^	42 (25.6)	2.62 ± 0.65			3.45 ± 0.37		
	≥50 ^d^	55 (33.5)	2.93 ± 0.50			3.49 ± 0.39		
		43.41 ± 11.37		0.28	<0.001		0.05	0.519
Education	College, University	153 (93.3)	2.69 ± 0.58	−0.87	0.383	3.45 ± 0.36	−0.88	0.379
	Graduate school or above	11 (6.7)	2.85 ± 0.59			3.55 ± 0.44		
Total work	<5	29 (17.7)	2.56 ± 0.60	2.70	0.048	3.47 ± 0.32	0.62	0.602
period (year)	5~<10	46 (28.0)	2.59 ± 0.57			3.47 ± 0.36		
	10~<20	53 (32.3)	2.73 ± 0.61			3.40 ± 0.38		
	≥20	36 (22.0)	2.90 ± 0.47			3.49 ± 0.38		
		12.62 ± 8.83		0.21	0.007		0.06	0.454
Work period	<3 ^a^	53 (32.3)	2.50 ± 0.56	4.66	0.004	3.43 ± 0.32	0.23	0.876
in community	3~<5 ^b^	23 (14.0)	2.74 ± 0.63		a < d	3.49 ± 0.36		
	5~<10 ^c^	34 (20.7)	2.66 ± 0.60			3.44 ± 0.38		
	≥10 ^d^	54 (32.9)	2.90 ± 0.49			3.47 ± 0.39		
		7.68 ± 7.30		0.24	0.002		0.06	0.423
Nursing experience for	Yes	78 (47.6)	2.84 ± 0.50	3.09	0.002	3.50 ± 0.35	1.68	0.095
cancer patients in treatment	No	86 (52.4)	2.57 ± 0.61			3.41 ± 0.37		
Experience of participating in	Yes	27 (16.5)	3.22 ± 0.52	−5.57	<0.001	3.48 ± 0.39	−0.31	0.755
cancer survivor care education	No	137 (83.5)	2.60 ± 0.53			3.45 ± 0.36		
Need for	Yes	163 (99.4)	2.71 ± 0.57	2.00	0.047	3.45 ± 0.36	0.26	0.798
cancer survivor care education	No	1 (0.6)	1.56 ± 0.00			3.36 ± 0.00		
Willingness to participate in	Yes	153 (93.3)	2.72 ± 0.56	1.65	0.101	3.45 ± 0.36	−1.06	0.293
cancer survivor care education	No	11 (6.7)	2.43 ± 0.69			3.56 ± 0.39		

‘a, b, c, d’ denote the groups distinguished in the ANOVA analysis.

**Table 2:** Difference between perceived knowledge and perceived importance regarding the cancer survivor nursing care (N = 164).

Categories	Contents	Knowledge	Importance	Discrepancy	t
		M ± SD	M ± SD	M ± SD	
Introduction to cancer survivors	1. Current status and key issues of cancer survivors	2.55 ± 0.77	3.38 ± 0.50	0.83 ± 0.80	13.35 ***
Physical problem	2. Complications of cancer treatment	2.76 ± 0.70	3.60 ± 0.49	0.84 ± 0.82	13.11 ***
	3. Management of comorbidities	2.87 ± 0.71	3.50 ± 0.50	0.63 ± 0.84	9.60 ***
	4. Fatigue	2.98 ± 0.62	3.34 ± 0.65	0.36 ± 0.80	6.14 ***
	5. Pain	3.01 ± 0.64	3.60 ± 0.56	0.60 ± 0.68	11.25 ***
	6. Sexual health	2.35 ± 0.78	2.90 ± 0.75	0.54 ± 0.82	8.43 ***
	7. Change in appearance	2.85 ± 0.70	3.33 ± 0.68	0.48 ± 0.73	8.45 ***
Rehabilitation	8. Physical rehabilitation after cancer treatment	2.57 ± 0.75	3.61 ± 0.50	1.04 ± 0.91	14.69 ***
Healthy lifestyle	9. Drinking	3.05 ± 0.69	3.52 ± 0.61	0.47 ± 0.77	7.80 ***
	10. Smoking	3.09 ± 0.69	3.58 ± 0.63	0.49 ± 0.79	7.96 ***
	11. Diet and nutrition	2.83 ± 0.72	3.65 ± 0.50	0.82 ± 0.84	12.50 ***
	12. Exercise	2.88 ± 0.73	3.64 ± 0.49	0.76 ± 0.83	11.79 ***
	13. Second primary cancer screening	2.71 ± 0.82	3.73 ± 0.46	1.02 ± 0.92	14.23 ***
	14. Vaccination	2.61 ± 0.83	3.26 ± 0.68	0.65 ± 0.90	9.31 ***
	15. Complementary therapy	2.43 ± 0.81	3.07 ± 0.75	0.64 ± 0.95	8.61 ***
Psychological	16. Distress(depression, anxiety, stress)	2.97 ± 0.68	3.68 ± 0.50	0.71 ± 0.77	11.81 ***
problem	17. Sleep disorder	2.92 ± 0.69	3.58 ± 0.53	0.66 ± 0.70	12.12 ***
	18. Cognitive decline	2.68 ± 0.77	3.43 ± 0.60	0.74 ± 0.84	11.33 ***
Social problem	19. Returning to work	2.63 ± 0.74	3.49 ± 0.59	0.86 ± 0.80	13.80 ***
	20. Economic support and related insurance systems	2.49 ± 0.80	3.60 ± 0.54	1.12 ± 0.92	15.49 ***
	21. Physical/mental problems of a cancer patient’s family	2.63 ± 0.74	3.49 ± 0.58	0.86 ± 0.80	13.42 ***
Spiritual problem	22. Religious activity	2.45 ± 0.83	3.10 ± 0.69	0.65 ± 0.81	10.19 ***
Search/learn	23. Provide training materials and information on cancer	2.60 ± 0.72	3.45 ± 0.52	0.85 ± 0.85	12.80 ***
Community network	24. Management of cancer survivors in the community	2.45 ± 0.84	3.45 ± 0.53	1.00 ± 1.01	12.69 ***
	25. Patient organization and related society	2.15 ± 0.78	3.34 ± 0.59	1.19 ± 0.95	16.03 ***

Discrepancy = Importance—Knowledge; *** *p* < 0.001.

**Table 3:** Priority contents according to the Borich needs assessment and the Locus for Focus models.

Categories	Contents	Borich Needs Assessment		Locus for Focus Model
		Needs Score	Ranking	(Quadrant)
Introduction to cancer survivors	1. Current status and key issues of cancer survivors	2.81	11	HL
Physical problem	2. Complications of cancer treatment	3.01	7	HH
	3. Management of comorbidities	2.20	16	LH
	4. Fatigue	1.20	25	LL
	5. Pain	2.15	17	LH
	6. Sexual health	1.57	24	LL
	7. Change in appearance	1.60	23	LL
Rehabilitation	8. Physical rehabilitation after cancer treatment	3.76	4	HH
Healthy lifestyle	9. Drinking	1.65	22	LH
	10. Smoking	1.77	21	LH
	11. Diet and nutrition	3.01	6	HH
	12. Exercise	2.77	12	HH
	13. Second primary cancer screening	3.79	3	HH
	14. Vaccination	2.13	18	LL
	15. Complementary therapy	1.97	20	LL
Psychological	16. Distress(depression, anxiety, stress)	2.60	13	LH
problem	17. Sleep disorder	2.36	15	LH
	18. Cognitive decline	2.55	14	LL
Social problem	19. Returning to work	3.00	8	HH
	20. Economic support and related insurance systems	4.02	1	HH
	21. Physical/mental problems of a cancer patient’s family	3.00	9	HH
Spiritual problem	22. Religious activity	2.00	19	LL
Search/learn	23. Provide training materials and information on cancer	2.93	10	HH
Community network	24. Management of cancer survivors in the community	3.45	5	HL
	25. Patient organization and related society	3.97	2	HL

HH, high discrepancy/high importance; HL, high discrepancy/low importance; LL, low discrepancy/low importance; LH, low discrepancy/high importance.

## Data Availability

The data presented in this study are available on request from the corresponding author. The datasets generated or analyzed during the current study are not publicly available due to ethical and privacy restrictions.

## Supplementary Materials

The following supporting information can be downloaded at: https://www.mdpi.com/article/10.3390/healthcare14142169/s1, Table S1: Difference between perceived knowledge and perceived importance regarding the cancer survivor nursing care according to educational experience; Table S2: Priority contents according to the Borich needs assessment and the Locus for Focus models.
