# Supplementary material for: Educational Needs Analysis for Enhancing Cancer Survivor Nursing Care of Community Nurses
Source: Healthcare (Basel). 2026 Jul 18;14(14):2169. doi: 10.3390/healthcare14142169 (PMC13411229; doi:10.3390/healthcare14142169)
Supplement: Supplementary file 1 [file healthcare-14-02169-s001.zip › healthcare-4399729-supplementary.pdf]

Supplementary Materials

Table S1. Difference between perceived knowledge and perceived importance regarding the cancer survivor nursing care according to educational experience

▣ Participants **with** prior educational experience in cancer survivor nursing (N=27)

Categories	Contents	Knowledge	Importance	Discrepancy	t
		M ± SD	M ± SD	M ± SD	
Introduction to cancer survivors	1. Current status and key issues of cancer survivors	3.19 ± 0.74	3.33 ± 0.48	0.15 ± 0.60	1.28
Physical problem	2. Complications of cancer treatment	3.33 ± 0.56	3.48 ± 0.51	0.15 ± 0.53	1.44
	3. Management of comorbidities	3.44 ± 0.58	3.48 ± 0.51	0.04 ± 0.52	0.37
	4. Fatigue	3.33 ± 0.56	3.52 ± 0.51	0.19 ± 0.40	2.43**
	5. Pain	3.37 ± 0.57	3.70 ± 0.47	0.33 ± 0.56	3.12**
	6. Sexual health	3.04 ± 0.65	3.19 ± 0.56	0.15 ± 0.36	2.13**
	7. Change in appearance	3.26 ± 0.59	3.44 ± 0.70	0.19 ± 0.48	1.99
Rehabilitation	8. Physical rehabilitation after cancer treatment	3.00 ± 0.83	3.56 ± 0.51	0.56 ± 0.89	3.24**
Healthy lifestyle	9. Drinking	3.44 ± 0.58	3.63 ± 0.49	0.19 ± 0.40	2.43**
	10. Smoking	3.44 ± 0.58	3.63 ± 0.49	0.19 ± 0.48	1.99
	11. Diet and nutrition	3.26 ± 0.53	3.63 ± 0.49	0.37 ± 0.57	3.41**
	12. Exercise	3.37 ± 0.57	3.63 ± 0.49	0.26 ± 0.66	2.05
	13. Second primary cancer screening	3.30 ± 0.67	3.67 ± 0.48	0.37 ± 0.69	2.80**
	14. Vaccination	3.22 ± 0.64	3.41 ± 0.57	0.19 ± 0.68	1.41
	15. Complementary therapy	2.96 ± 0.76	3.26 ± 0.71	0.30 ± 0.61	2.53**
Psychological problem	16. Distress(depression, anxiety, stress)	3.41 ± 0.57	3.63 ± 0.49	0.22 ± 0.58	2.00
	17. Sleep disorder	3.33 ± 0.68	3.63 ± 0.49	0.30 ± 0.72	2.13**
	18. Cognitive decline	3.22 ± 0.75	3.52 ± 0.58	0.30 ± 0.67	2.30**
Social problem	19. Returning to work	3.15 ± 0.66	3.44 ± 0.58	0.30 ± 0.54	2.84**
	20. Economic support and related insurance systems	3.04 ± 0.76	3.33 ± 0.62	0.30 ± 0.72	2.13**

	21. Physical/mental problems of a cancer patient's family	3.15 ± 0.66	3.30 ± 0.67	0.15 ± 0.46	1.69
Spiritual problem	22. Religious activity	3.04 ± 0.71	3.37 ± 0.57	0.33 ± 0.48	3.61**
Search/learn	23. Provide training materials and information on cancer	3.19± 0.56	3.41 ± 0.50	0.22 ± 0.51	2.28**
Community network	24. Management of cancer survivors in the community	3.22 ± 0.58	3.37 ± 0.57	0.15 ± 0.53	1.44
	25. Patient organization and related society	2.78 ± 0.80	3.26 ± 0.59	0.48 ± 0.75	3.23**

Discrepancy=Importance - Knowledge; ***p*<.05

▣ Participants **without** prior educational experience in cancer survivor nursing (N=137)

Categories	Contents	Knowledge	Importance	Discrepancy	t
		M ± SD	M ± SD	M ± SD	
Introduction to cancer survivors	1. Current status and key issues of cancer survivors	2.43 ± 0.72	3.39 ± 0.51	0.96 ± 0.76	14.81***
Physical problem	2. Complications of cancer treatment	2.65 ± 0.67	3.62 ± 0.49	0.97 ± 0.80	14.30***
	3. Management of comorbidities	2.76 ± 0.68	3.50 ± 0.50	0.75 ± 0.84	10.37***
	4. Fatigue	2.91 ± 0.61	3.31 ± 0.67	0.39 ± 0.80	5.78***
	5. Pain	2.93 ± 0.63	3.58 ± 0.58	0.65 ± 0.69	10.98***
	6. Sexual health	2.22 ± 0.74	2.84 ± 0.77	0.62 ± 0.87	8.38***
	7. Change in appearance	2.77 ± 0.67	3.31 ± 0.68	0.54 ± 0.76	8.35***
Rehabilitation	8. Physical rehabilitation after cancer treatment	2.48 ± 0.71	3.62 ± 0.50	1.14 ± 0.88	15.07***
Healthy lifestyle	9. Drinking	2.98 ± 0.68	3.50 ± 0.63	0.53 ± 0.81	7.56***
	10. Smoking	3.01 ± 0.69	3.57 ± 0.65	0.56 ± 0.83	7.82***
	11. Diet and nutrition	2.74 ± 0.72	3.66 ± 0.51	0.91 ± 0.86	12.40***
	12. Exercise	2.78 ± 0.73	3.64 ± 0.50	0.86 ± 0.82	12.23***
	13. Second primary cancer screening	2.59 ± 0.80	3.74 ± 0.46	1.15 ± 0.90	14.84***
	14. Vaccination	2.49 ± 0.81	3.23 ± 0.70	0.75 ± 0.91	9.60***
	15. Complementary therapy	2.33 ± 0.78	3.04 ± 0.75	0.71 ± 0.99	8.34***
Psychological problem	16. Distress(depression, anxiety, stress)	2.88 ± 0.67	3.69 ± 0.50	0.80 ± 0.77	12.28***
	17. Sleep disorder	2.84 ± 0.67	3.57 ± 0.54	0.73 ± 0.67	12.76***
	18. Cognitive decline	2.58 ± 0.73	3.41 ± 0.60	0.83 ± 0.85	11.52***
Social problem	19. Returning to work	2.53 ± 0.71	3.50 ± 0.60	0.97 ± 0.80	14.30***
	20. Economic support and related insurance systems	2.38 ± 0.76	3.66 ± 0.51	1.28 ± 0.87	17.14***
	21. Physical/mental problems of a cancer patient's family	2.53 ± 0.72	3.53 ± 0.56	1.00 ± 0.80	14.55***
Spiritual problem	22. Religious activity	2.34 ± 0.70	3.04 ± 0.70	0.71 ± 0.85	9.75***
Search/learn	23. Provide training materials and information on cancer	2.49± 0.70	3.46 ± 0.53	0.97 ± 0.85	13.39***
Community network	24. Management of cancer survivors in the community	2.29 ± 0.80	3.46 ± 0.53	1.17 ± 1.00	13.71***
	25. Patient organization and related society	2.02 ± 0.71	3.35 ± 0.59	1.33 ± 0.92	16.82***

Discrepancy=Importance - Knowledge; ***p<.001

Table S2. Priority contents according to the Borich needs assessment and the Locus for Focus models.

▣ Participants **with** prior educational experience in cancer survivor nursing

Categories	Contents	Borich needs assessment		Locus for Focus model
		Needs score	Ranking	(Quadrant)
Introduction to cancer survivors	1. Current status and key issues of cancer survivors	0.49	22	LL
Physical problem	2. Complications of cancer treatment	0.52	20	LH
	3. Management of comorbidities	0.13	25	LH
	4. Fatigue	0.65	17	LH
	5. Pain	1.23	5	HH
	6. Sexual health	0.47	24	LL
	7. Change in appearance	0.64	18	LL
Rehabilitation	8. Physical rehabilitation after cancer treatment	1.98	1	HH
Healthy lifestyle	9. Drinking	0.67	15	LH
	10. Smoking	0.67	15	LH
	11. Diet and nutrition	1.34	4	HH
	12. Exercise	0.94	12	HH
	13. Second primary cancer screening	1.36	3	HH
	14. Vaccination	0.63	19	LL
	15. Complementary therapy	0.97	11	HL
Psychological problem	16. Distress(depression, anxiety, stress)	0.81	13	LH
	17. Sleep disorder	1.08	7	HH
	18. Cognitive decline	1.04	8	HH
Social problem	19. Returning to work	1.02	9	HL
	20. Economic support and related insurance systems	0.99	10	HL
	21. Physical/mental problems of a cancer patient's family	0.49	23	LL
Spiritual problem	22. Religious activity	1.12	6	HL
Search/learn	23. Provide training materials and information on cancer	0.76	14	LL
Community network	24. Management of cancer survivors in the community	0.50	21	LL
	25. Patient organization and related society	1.57	2	HL

▣ Participants **without** prior educational experience in cancer survivor nursing

Categories	Contents	Borich needs assessment		Locus for Focus model
		Needs score	Ranking	(Quadrant)
Introduction to cancer survivors	1. Current status and key issues of cancer survivors	3.27	11	HL
Physical problem	2. Complications of cancer treatment	3.51	7	HH
	3. Management of comorbidities	2.61	15	LH
	4. Fatigue	1.30	25	LL
	5. Pain	2.33	18	LH
	6. Sexual health	1.76	24	LL
	7. Change in appearance	1.79	23	LL
Rehabilitation	8. Physical rehabilitation after cancer treatment	4.12	4	HH
Healthy lifestyle	9. Drinking	1.84	22	LH
	10. Smoking	1.98	21	LH
	11. Diet and nutrition	3.34	10	HH
	12. Exercise	3.14	12	HH
	13. Second primary cancer screening	4.28	3	HH
	14. Vaccination	2.41	17	LL
	15. Complementary therapy	2.15	20	LL
Psychological problem	16. Distress(depression, anxiety, stress)	2.96	13	LH
	17. Sleep disorder	2.61	16	LH
	18. Cognitive decline	2.84	14	LL
Social problem	19. Returning to work	3.40	8	HH
	20. Economic support and related insurance systems	4.67	1	HH
	21. Physical/mental problems of a cancer patient's family	3.53	6	HH
Spiritual problem	22. Religious activity	2.16	19	LL
Search/learn	23. Provide training materials and information on cancer	3.36	9	HH
Community network	24. Management of cancer survivors in the community	4.04	5	HH
	25. Patient organization and related society	4.45	2	HL
